# 2-Chloro-*N*-(3,4-dimethyl­phen­yl)benzamide

**DOI:** 10.1107/S1600536811027267

**Published:** 2011-07-13

**Authors:** Vinola Z. Rodrigues, Sabine Foro, B. Thimme Gowda

**Affiliations:** a Department of Chemistry, Mangalore University, Mangalagangotri 574 199, Mangalore, India; b Institute of Materials Science, Darmstadt University of Technology, Petersenstrasse 23, D-64287 Darmstadt, Germany

## Abstract

In the title compound, C_15_H_14_ClNO, the conformation of the N—H bond is *anti* to the *meta*-methyl group in the aniline ring, while that of the C=O bond is *anti* to the *ortho*-chloro group in the benzoyl ring. The mean planes through the two benzene rings make a dihedral angle of 80.8 (2)°. In the crystal, mol­ecules are linked by inter­molecular N—H⋯O hydrogen bonds, forming column-like chains along the *b* axis.

## Related literature

For the preparation of the title compound, see: Gowda *et al.* (2003 ▶). For our studies on the effects of substituents on the structures of *N*-(ar­yl)-amides, see: Bhat & Gowda (2000 ▶); Gowda *et al.* (2007 ▶) and on *N*-(ar­yl)-benzamides, see: Gowda *et al.* (2009 ▶); Gowda *et al.* (2010 ▶). For related structure, see: Bowes *et al.* (2003 ▶).
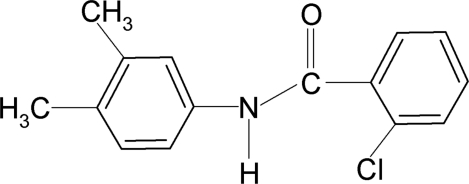


## Experimental

### 

#### Crystal data

C_15_H_14_ClNO
*M* *_r_* = 259.72
Monoclinic, 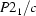

*a* = 20.893 (2) Å
*b* = 7.259 (1) Å
*c* = 8.970 (1) Å
β = 91.95 (1)°
*V* = 1359.6 (3) Å^3^
*Z* = 4
Mo *K*α radiation
μ = 0.27 mm^−1^
*T* = 293 K
0.30 × 0.26 × 0.16 mm

#### Data collection

Oxford Diffraction Xcalibur diffractometer with a Sapphire CCD detector
Absorption correction: multi-scan (*CrysAlis RED*; Oxford Diffraction, 2009 ▶) *T*_min_ = 0.924, *T*_max_ = 0.958
4865 measured reflections
2489 independent reflections
1570 reflections with *I* > 2σ(*I*)
*R*_int_ = 0.027

#### Refinement

*R*[*F* ^2^ > 2σ(*F* ^2^)] = 0.076
*wR*(*F* ^2^) = 0.217
*S* = 1.10
2489 reflections
168 parameters
1 restraint
H atoms treated by a mixture of independent and constrained refinement
Δρ_max_ = 0.32 e Å^−3^
Δρ_min_ = −0.28 e Å^−3^

### 

Data collection: *CrysAlis CCD* (Oxford Diffraction, 2009 ▶); cell refinement: *CrysAlis RED* (Oxford Diffraction, 2009 ▶); data reduction: *CrysAlis RED*; program(s) used to solve structure: *SHELXS97* (Sheldrick, 2008 ▶); program(s) used to refine structure: *SHELXL97* (Sheldrick, 2008 ▶); molecular graphics: *PLATON* (Spek, 2009 ▶); software used to prepare material for publication: *SHELXL97*.

## Supplementary Material

Crystal structure: contains datablock(s) I, global. DOI: 10.1107/S1600536811027267/nc2237sup1.cif

Structure factors: contains datablock(s) I. DOI: 10.1107/S1600536811027267/nc2237Isup2.hkl

Supplementary material file. DOI: 10.1107/S1600536811027267/nc2237Isup3.cml

Additional supplementary materials:  crystallographic information; 3D view; checkCIF report

## Figures and Tables

**Table 1:** Hydrogen-bond geometry (Å, °)

*D*—H⋯*A*	*D*—H	H⋯*A*	*D*⋯*A*	*D*—H⋯*A*
N1—H1*N*⋯O1^i^	0.87 (2)	2.00 (2)	2.850 (4)	168 (4)

Symmetry code: (i) 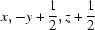.

**Fig. 1.**
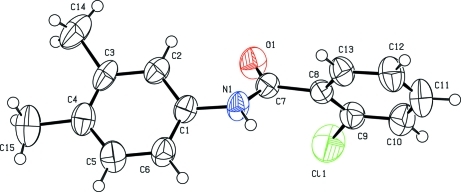
Molecular structure of (I) showing the atom labelling scheme. Displacement ellipsoids are drawn at the 30% probability level and H atoms are represented as small spheres of arbitrary radii.

**Fig. 2.**
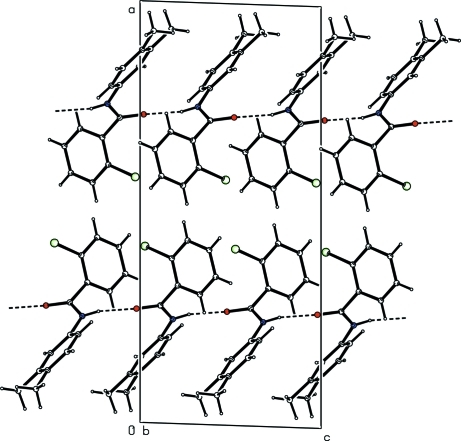
View of the crystal packing of (I), showing the chains of molecules linked by intermolecular N–H···O hydrogen bonds (dashed lines).

## supplementary crystallographic information

### Comment

To explore the effect of substituents on the structures of acetanilides (Bhat & Gowda, 2000; Gowda, Foro *et al.*, 2007) and benzanilides (Gowda, Foro *et al.*, 2009; Gowda, Jyothi *et al.*, 2003; Gowda, Tokarčík *et al.*, 2010), in the present work, the structure of 2-chloro-*N*-(3,4-dimethylphenyl)-benzamide (I) has been determined. The N—H and C═O bonds in the amide group are *anti* to each other (Fig.1), similar to that observed in *N*-(phenyl)-benzamide (Bowes *et al.*, 2003), 2-chloro-*N*-(phenyl)-benzamide (Gowda, Jyothi *et al.*, 2003), 2-chloro-*N*-(2,3-dimethylphenyl)- benzamide (Gowda, Tokarčík *et al.*, 2010) and 2-chloro-*N*- (3,5-dimethylphenyl)-benzamide (Gowda, Foro *et al.*, 2009).

The conformation of the N—H bond is *anti* to the *meta*-methyl group in the aniline ring, while that of the C=O bond is *anti* to the *ortho*-chloro group in the benzoyl ring.

The central amide group –NHCO– is inclined to the benzoyl ring with C9 – C8 – C7 – N1 and C9 – C8 – C7 – N1 torsional angles of 120.2 (5) and -62.7 (5)°, respectively, while it is inclined to the aniline benzene ring with C2 – C1 – N1 – C7 and C6 – C1 – N1 – C7 torsional angles of -40.1 (7) and 139.5 (7)°, respectively.

The mean planes through the two benzene rings make dihedral angle of 80.8 (2)°. The intermolecular N–H···O hydrogen bonds (Table 1) link the molecules into column like chains extending along the *b* axis (Fig. 2).

### Experimental

The title compound was prepared according to the literature method (Gowda Jyothi *et al.*, 2003). The purity of the compound was checked by determining its melting point. It was characterized by recording its infrared and NMR spectra. Prism like light brown single crystals of the title compound were obtained from an ethanolic solution and used for X-ray diffraction studies at room temperature.

### Refinement

The H atom of the NH group was located in a difference map and later restrained to N—H = 0.86 (2) %A. The other H atoms were positioned with idealized geometry using a riding model with the aromatic C—H = 0.93 Å and the methyl C—H = 0.96 Å. All H atoms were refined with isotropic displacement parameters. The *U*_iso_(H) values were set at 1.2*U*_eq_(C aromatic, N) and 1.5*U*_eq_(C methyl).

### Crystal data

**Table:**

C_15_H_14_ClNO	*F*(000) = 544
*M**_r_* = 259.72	*D*_x_ = 1.269 Mg m^−^^3^
Monoclinic, *P*2_1_/*c*	Mo *K*α radiation, λ = 0.71073 Å
Hall symbol: -P 2ybc	Cell parameters from 872 reflections
*a* = 20.893 (2) Å	θ = 2.8–27.9°
*b* = 7.259 (1) Å	µ = 0.27 mm^−^^1^
*c* = 8.970 (1) Å	*T* = 293 K
β = 91.95 (1)°	Prism, light brown
*V* = 1359.6 (3) Å^3^	0.30 × 0.26 × 0.16 mm
*Z* = 4	

### Data collection

**Table:**

Oxford Diffraction Xcalibur diffractometer with a Sapphire CCD detector	2489 independent reflections
Radiation source: fine-focus sealed tube	1570 reflections with *I* > 2σ(*I*)
graphite	*R*_int_ = 0.027
Rotation method data acquisition using ω scans	θ_max_ = 25.4°, θ_min_ = 2.9°
Absorption correction: multi-scan (*CrysAlis RED*; Oxford Diffraction, 2009)	*h* = −25→16
*T*_min_ = 0.924, *T*_max_ = 0.958	*k* = −8→8
4865 measured reflections	*l* = −10→10

### Refinement

**Table:**

Refinement on *F*^2^	Primary atom site location: structure-invariant direct methods
Least-squares matrix: full	Secondary atom site location: difference Fourier map
*R*[*F*^2^ > 2σ(*F*^2^)] = 0.076	Hydrogen site location: inferred from neighbouring sites
*wR*(*F*^2^) = 0.217	H atoms treated by a mixture of independent and constrained refinement
*S* = 1.10	*w* = 1/[σ^2^(*F*_o_^2^) + (0.074*P*)^2^ + 2.1954*P*] where *P* = (*F*_o_^2^ + 2*F*_c_^2^)/3
2489 reflections	(Δ/σ)_max_ < 0.001
168 parameters	Δρ_max_ = 0.32 e Å^−^^3^
1 restraint	Δρ_min_ = −0.28 e Å^−^^3^

### Special details

**Table:**

Experimental. CrysAlis RED (Oxford Diffraction, 2009) Empirical absorption correction using spherical harmonics, implemented in SCALE3 ABSPACK scaling algorithm.
Geometry. All e.s.d.'s (except the e.s.d. in the dihedral angle between two l.s. planes) are estimated using the full covariance matrix. The cell e.s.d.'s are taken into account individually in the estimation of e.s.d.'s in distances, angles and torsion angles; correlations between e.s.d.'s in cell parameters are only used when they are defined by crystal symmetry. An approximate (isotropic) treatment of cell e.s.d.'s is used for estimating e.s.d.'s involving l.s. planes.
Refinement. Refinement of *F*^2^ against ALL reflections. The weighted *R*-factor *wR* and goodness of fit *S* are based on *F*^2^, conventional *R*-factors *R* are based on *F*, with *F* set to zero for negative *F*^2^. The threshold expression of *F*^2^ > σ(*F*^2^) is used only for calculating *R*-factors(gt) *etc*. and is not relevant to the choice of reflections for refinement. *R*-factors based on *F*^2^ are statistically about twice as large as those based on *F*, and *R*- factors based on ALL data will be even larger.

### Fractional atomic coordinates and isotropic or equivalent isotropic displacement parameters (Å^2^)

**Table:**

	*x*	*y*	*z*	*U*_iso_*/*U*_eq_	
C1	0.2020 (2)	0.0975 (6)	0.1139 (4)	0.0465 (11)	
C2	0.15704 (19)	0.1524 (7)	0.0060 (4)	0.0509 (11)	
H2	0.1555	0.2746	−0.0251	0.061*	
C3	0.11372 (19)	0.0239 (8)	−0.0568 (4)	0.0549 (13)	
C4	0.1162 (2)	−0.1594 (7)	−0.0111 (5)	0.0571 (13)	
C5	0.1615 (2)	−0.2089 (8)	0.0966 (5)	0.0629 (13)	
H5	0.1635	−0.3308	0.1283	0.075*	
C6	0.2037 (2)	−0.0836 (7)	0.1584 (5)	0.0572 (12)	
H6	0.2338	−0.1215	0.2309	0.069*	
C7	0.2788 (2)	0.3563 (6)	0.1115 (4)	0.0443 (10)	
C8	0.3274 (2)	0.4554 (6)	0.2085 (4)	0.0446 (10)	
C9	0.3914 (2)	0.4586 (6)	0.1757 (5)	0.0545 (12)	
C10	0.4350 (2)	0.5547 (8)	0.2628 (6)	0.0706 (14)	
H10	0.4782	0.5528	0.2414	0.085*	
C11	0.4141 (3)	0.6539 (8)	0.3824 (6)	0.0832 (18)	
H11	0.4433	0.7225	0.4398	0.100*	
C12	0.3509 (3)	0.6532 (8)	0.4181 (6)	0.0733 (15)	
H12	0.3372	0.7191	0.5000	0.088*	
C13	0.3083 (2)	0.5538 (7)	0.3309 (5)	0.0566 (12)	
H13	0.2653	0.5526	0.3548	0.068*	
C14	0.0649 (2)	0.0927 (9)	−0.1727 (6)	0.0855 (19)	
H14A	0.0722	0.2210	−0.1915	0.103*	
H14B	0.0225	0.0762	−0.1365	0.103*	
H14C	0.0690	0.0243	−0.2634	0.103*	
C15	0.0705 (3)	−0.3013 (9)	−0.0752 (6)	0.0836 (18)	
H15A	0.0740	−0.3054	−0.1816	0.100*	
H15B	0.0275	−0.2691	−0.0511	0.100*	
H15C	0.0810	−0.4199	−0.0337	0.100*	
N1	0.24717 (17)	0.2223 (5)	0.1808 (3)	0.0506 (10)	
H1N	0.260 (2)	0.189 (6)	0.270 (3)	0.061*	
O1	0.26938 (16)	0.3992 (5)	−0.0198 (3)	0.0633 (9)	
Cl1	0.41840 (7)	0.3313 (2)	0.02704 (17)	0.0920 (6)	

### Atomic displacement parameters (Å^2^)

**Table:**

	*U*^11^	*U*^22^	*U*^33^	*U*^12^	*U*^13^	*U*^23^
C1	0.047 (2)	0.061 (3)	0.031 (2)	−0.005 (2)	−0.0019 (17)	−0.0008 (19)
C2	0.047 (2)	0.064 (3)	0.042 (2)	0.003 (2)	−0.0029 (18)	−0.002 (2)
C3	0.038 (2)	0.089 (4)	0.037 (2)	0.001 (2)	−0.0061 (17)	−0.010 (2)
C4	0.052 (3)	0.076 (4)	0.044 (2)	−0.014 (3)	0.0045 (19)	−0.009 (2)
C5	0.069 (3)	0.064 (3)	0.055 (3)	−0.015 (3)	−0.002 (2)	0.002 (2)
C6	0.058 (3)	0.065 (3)	0.048 (2)	−0.011 (2)	−0.010 (2)	0.005 (2)
C7	0.051 (2)	0.049 (3)	0.032 (2)	−0.001 (2)	−0.0033 (17)	−0.0021 (19)
C8	0.055 (3)	0.040 (2)	0.037 (2)	−0.003 (2)	−0.0077 (18)	0.0038 (18)
C9	0.058 (3)	0.052 (3)	0.053 (3)	−0.003 (2)	−0.003 (2)	−0.003 (2)
C10	0.056 (3)	0.077 (4)	0.079 (4)	−0.013 (3)	−0.002 (3)	−0.001 (3)
C11	0.082 (4)	0.083 (4)	0.083 (4)	−0.029 (3)	−0.012 (3)	−0.022 (3)
C12	0.084 (4)	0.074 (4)	0.062 (3)	−0.013 (3)	0.000 (3)	−0.025 (3)
C13	0.068 (3)	0.055 (3)	0.047 (2)	−0.006 (2)	0.000 (2)	−0.010 (2)
C14	0.064 (3)	0.127 (5)	0.064 (3)	0.011 (3)	−0.024 (3)	−0.009 (3)
C15	0.079 (4)	0.107 (5)	0.064 (3)	−0.034 (4)	0.000 (3)	−0.019 (3)
N1	0.059 (2)	0.060 (2)	0.0313 (17)	−0.015 (2)	−0.0121 (16)	0.0037 (17)
O1	0.082 (2)	0.073 (2)	0.0339 (16)	−0.0139 (18)	−0.0117 (14)	0.0075 (15)
Cl1	0.0697 (9)	0.1153 (14)	0.0917 (11)	0.0070 (9)	0.0146 (7)	−0.0370 (9)

### Geometric parameters (Å, °)

**Table:**

C1—C6	1.375 (6)	C9—C10	1.371 (6)
C1—C2	1.384 (6)	C9—Cl1	1.732 (5)
C1—N1	1.426 (5)	C10—C11	1.375 (7)
C2—C3	1.404 (6)	C10—H10	0.9300
C2—H2	0.9300	C11—C12	1.369 (7)
C3—C4	1.393 (7)	C11—H11	0.9300
C3—C14	1.516 (6)	C12—C13	1.370 (7)
C4—C5	1.377 (7)	C12—H12	0.9300
C4—C15	1.504 (7)	C13—H13	0.9300
C5—C6	1.371 (6)	C14—H14A	0.9600
C5—H5	0.9300	C14—H14B	0.9600
C6—H6	0.9300	C14—H14C	0.9600
C7—O1	1.228 (4)	C15—H15A	0.9600
C7—N1	1.340 (5)	C15—H15B	0.9600
C7—C8	1.500 (5)	C15—H15C	0.9600
C8—C9	1.379 (6)	N1—H1N	0.866 (19)
C8—C13	1.380 (6)		
			
C6—C1—C2	119.3 (4)	C9—C10—C11	119.3 (5)
C6—C1—N1	118.3 (4)	C9—C10—H10	120.4
C2—C1—N1	122.4 (4)	C11—C10—H10	120.4
C1—C2—C3	120.1 (5)	C12—C11—C10	121.0 (5)
C1—C2—H2	119.9	C12—C11—H11	119.5
C3—C2—H2	119.9	C10—C11—H11	119.5
C4—C3—C2	120.0 (4)	C11—C12—C13	118.8 (5)
C4—C3—C14	122.2 (5)	C11—C12—H12	120.6
C2—C3—C14	117.8 (5)	C13—C12—H12	120.6
C5—C4—C3	118.2 (4)	C12—C13—C8	121.8 (5)
C5—C4—C15	120.1 (5)	C12—C13—H13	119.1
C3—C4—C15	121.7 (4)	C8—C13—H13	119.1
C6—C5—C4	121.9 (5)	C3—C14—H14A	109.5
C6—C5—H5	119.0	C3—C14—H14B	109.5
C4—C5—H5	119.0	H14A—C14—H14B	109.5
C5—C6—C1	120.4 (4)	C3—C14—H14C	109.5
C5—C6—H6	119.8	H14A—C14—H14C	109.5
C1—C6—H6	119.8	H14B—C14—H14C	109.5
O1—C7—N1	124.3 (4)	C4—C15—H15A	109.5
O1—C7—C8	121.2 (4)	C4—C15—H15B	109.5
N1—C7—C8	114.5 (3)	H15A—C15—H15B	109.5
C9—C8—C13	118.1 (4)	C4—C15—H15C	109.5
C9—C8—C7	121.8 (4)	H15A—C15—H15C	109.5
C13—C8—C7	120.1 (4)	H15B—C15—H15C	109.5
C10—C9—C8	121.1 (4)	C7—N1—C1	126.6 (3)
C10—C9—Cl1	118.9 (4)	C7—N1—H1N	119 (3)
C8—C9—Cl1	119.9 (3)	C1—N1—H1N	113 (3)
			
C6—C1—C2—C3	0.1 (6)	N1—C7—C8—C13	−62.7 (5)
N1—C1—C2—C3	179.8 (4)	C13—C8—C9—C10	0.8 (7)
C1—C2—C3—C4	−0.4 (6)	C7—C8—C9—C10	177.9 (4)
C1—C2—C3—C14	179.0 (4)	C13—C8—C9—Cl1	177.8 (3)
C2—C3—C4—C5	0.5 (6)	C7—C8—C9—Cl1	−5.0 (6)
C14—C3—C4—C5	−178.9 (4)	C8—C9—C10—C11	−2.0 (8)
C2—C3—C4—C15	179.8 (4)	Cl1—C9—C10—C11	−179.1 (4)
C14—C3—C4—C15	0.4 (7)	C9—C10—C11—C12	2.1 (9)
C3—C4—C5—C6	−0.3 (7)	C10—C11—C12—C13	−1.0 (9)
C15—C4—C5—C6	−179.6 (5)	C11—C12—C13—C8	−0.2 (8)
C4—C5—C6—C1	0.0 (7)	C9—C8—C13—C12	0.3 (7)
C2—C1—C6—C5	0.1 (7)	C7—C8—C13—C12	−176.9 (5)
N1—C1—C6—C5	−179.6 (4)	O1—C7—N1—C1	5.7 (7)
O1—C7—C8—C9	−60.5 (6)	C8—C7—N1—C1	−175.1 (4)
N1—C7—C8—C9	120.2 (5)	C6—C1—N1—C7	139.5 (5)
O1—C7—C8—C13	116.5 (5)	C2—C1—N1—C7	−40.1 (7)

### Hydrogen-bond geometry (Å, °)

**Table:**

*D*—H···*A*	*D*—H	H···*A*	*D*···*A*	*D*—H···*A*
N1—H1N···O1^i^	0.87 (2)	2.00 (2)	2.850 (4)	168 (4)

Symmetry codes: (i) *x*, −*y*+1/2, *z*+1/2.
